# KInhibition: A Kinase Inhibitor Selection Portal

**DOI:** 10.1016/j.isci.2018.09.009

**Published:** 2018-09-18

**Authors:** Thomas Bello, Taranjit S. Gujral

**Affiliations:** 1 Human Biology Division, Fred Hutchinson Cancer Research Center, Seattle, WA 98109, USA; 2 Department of Molecular and Cellular Biology, University of Washington, Seattle, WA 98195-7275, USA

**Keywords:** Molecular Biology, Bioinformatics, Software Engineering

## Abstract

Protein kinases constitute a large class of signaling molecules frequently targeted in research and clinical uses. However, kinase inhibitors are notoriously non-specific, making it difficult to select an appropriate inhibitor for a given kinase. Available data from large-scale kinase inhibitor screens are often difficult to query. Here, we present KInhibition (https://kinhibition.fredhutch.org), an online portal that allows users to search publicly available datasets to find selective inhibitors for a chosen kinase or group of kinases. Compounds are sorted by a KInhibition Selectivity Score, calculated based on compounds' activity against the selected kinase(s) versus activity against all other kinases for which that compound has been profiled. The current version allows users to query four datasets, with a framework that can easily accommodate additional datasets. KInhibition represents a powerful platform through which researchers from broad areas of biology, chemistry, and pharmacology can easily interrogate large datasets to help guide their selection of kinase inhibitors.

## Highlights

• An easy-to-use tool that compiles datasets from kinase inhibitor screens
• A KInhibition Selectivity Score quantifies each compound's selectivity
• Users can select kinases, view compounds and selectivity, and download the results
• KInhibition is broadly applicable across life science disciplines

## Graphical Abstract

**Figure:**
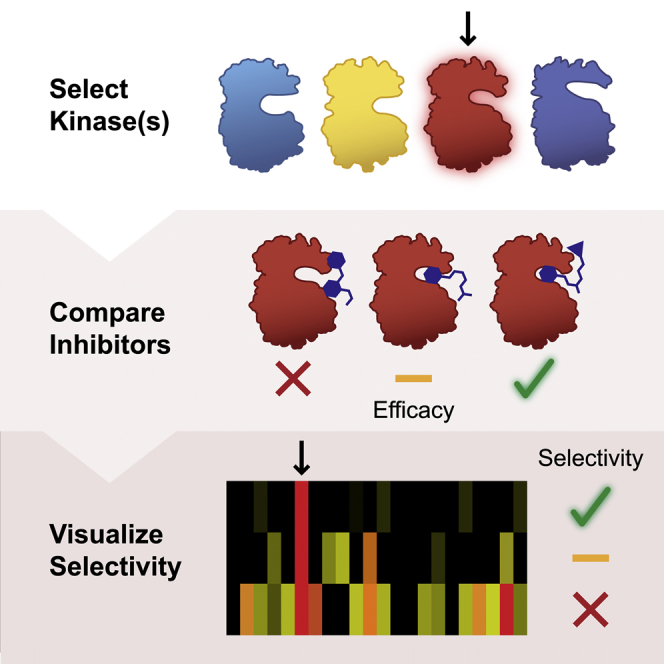

## Introduction

A fundamental aspect of cell biology is the study of cellular signaling, the process by which cells sense their surroundings, respond to environmental cues, and transfer information (Downward, 2001). Both clinicians and researchers rely on the ability to selectively perturb the function of specific signaling molecules, often by using small molecule inhibitors (Jin et al., 2014). In particular, kinases represent a large class of proteins that are key mediators of signaling pathways and important targets for research and therapy (Wu et al., 2015). In the last 10 years alone, there have been more than 1.5 million publications on kinases, and countless small-molecule inhibition studies spanning the majority of the >500 kinases in the human kinome (Wu et al., 2015), underscoring the central role of kinase signaling and inhibition in molecular and cellular biology.

Despite the enormity of research that has been done on kinase signaling, there remains a confounding challenge when attempting to selectively inhibit a desired molecule. Mainly due to the high structural similarity among kinases, nearly all available small-molecule kinase inhibitors exhibit some promiscuity, causing undesired “off-target” effects (Anastassiadis et al., 2011, Bain et al., 2007, Davis et al., 2011, Karaman et al., 2008). Even many compounds described as being “specific” or “selective” have confounding off-target effects, making the selection, use, and analysis of the appropriate kinase inhibitor difficult. A number of large-scale kinase inhibitor screens have been undertaken in an attempt to quantify these effects (Anastassiadis et al., 2011, Dranchak et al., 2013, Duong-Ly et al., 2016, Gao et al., 2013, Klaeger et al., 2017, Koleti et al., 2017), and many of these results are publicly available. However, these data are decentralized and difficult to query, with results often being spread across multiple data files that must be downloaded and opened individually. Furthermore, there are multiple methods employed for representing or quantifying “selectivity” (Anastassiadis et al., 2011, Cheng et al., 2010, Davis et al., 2011, Graczyk, 2007, Karaman et al., 2008, Klaeger et al., 2017, Uitdehaag and Zaman, 2011), which may yield conflicting results. Thus, the ultimate challenge of identifying the right kinase inhibitor for a biological task remains unresolved. Here we present KInhibition, a powerful platform tool that allows researchers to search through multiple kinase inhibitor screens, visualize the relevant data, and choose the most selective and appropriate kinase inhibitor for the task at hand. We anticipate that KInhibition will be adopted by the broader research community equivalent to RNAi target sequence or CRISPR guide RNA selection tools.

## Results

### The Theory behind KInhibition

KInhibition is a platform tool designed to answer the question “Which compound should be used to inhibit a chosen kinase or pathway?” The first, but often the most critical, step in answering this question is to locate and format the relevant data from the publicly available kinase inhibitor screens. These datasets may be initially found in somewhat intractable formats, but nearly all of them can be summarized by a matrix of drug-target interactions, with rows representing the compounds tested, columns representing the kinases screened, and entries being that compound's effect on that kinase. The main requirement for a dataset to be included in KInhibition is for it to be formatted in this manner, making the addition of future datasets to this platform a relatively trivial task.

A unified data format allows us to address the more nuanced issue of how to quantify the “selectivity” of a given inhibitor. Although there have been many proposed metrics for selectivity (Anastassiadis et al., 2011, Cheng et al., 2010, Graczyk, 2007, Klaeger et al., 2017, Uitdehaag and Zaman, 2011), very few are computationally robust enough to apply across datasets. We developed a “KInhibition Selectivity Score,” which quantifies the selectivity of a compound based on its on-target inhibition (“inhibition score”) and its off-target effects (“inhibition penalty”). The inhibition score is simply the inhibition of the selected kinase, or a geometric mean if multiple kinases are chosen. The inhibition penalty is further divided into two sub-penalties. The first quantifies the broad inhibition activity of a compound, and will therefore best account for the extreme case in which a compound inhibits nearly every kinase tested, but to a small degree (e.g., 10% of control) compared with the intended target. The second sub-penalty specifically quantifies off-target effects that are close in magnitude to the inhibition of the intended target. This accounts for the other extreme case, in which a compound inhibits only a few (e.g., 2–10) off-target kinases, but with a magnitude comparable to or greater than the intended target. Both these extremes represent distinct types of off-target effects that must be considered when choosing the appropriate kinase inhibitor for an experiment.

The KInhibition Selectivity Score has numerous advantages that give the user the most relevant information and the most control over the decision of which compound to use. First, it is designed to work with percent-of-control data, rather than binding coefficients (K_i_) or IC_50_ values, like previously reported partition indices or entropy-based scores (Uitdehaag and Zaman, 2011). This allows it to be used for screens performed even at a single dose, treating additional doses as separate compounds or entries in the matrix. Second, this score quantifies selectivity for a user-defined set of up to 10 “on-target” kinases, rather than simply basing all calculations on the most inhibited kinase for each compound. Third, as previously mentioned, this score accounts for both the number and the magnitude of off-target effects better than previous scores (such as the Gini coefficient), allowing researchers to select the inhibitor most suited to their needs. Finally, this scoring metric does not rely on any hard-coded values, arbitrary thresholds, or data binning, giving it an advantage over S(x) scores or Ambit scores (Cheng et al., 2010).

### Using KInhibition

**Figure 1:**
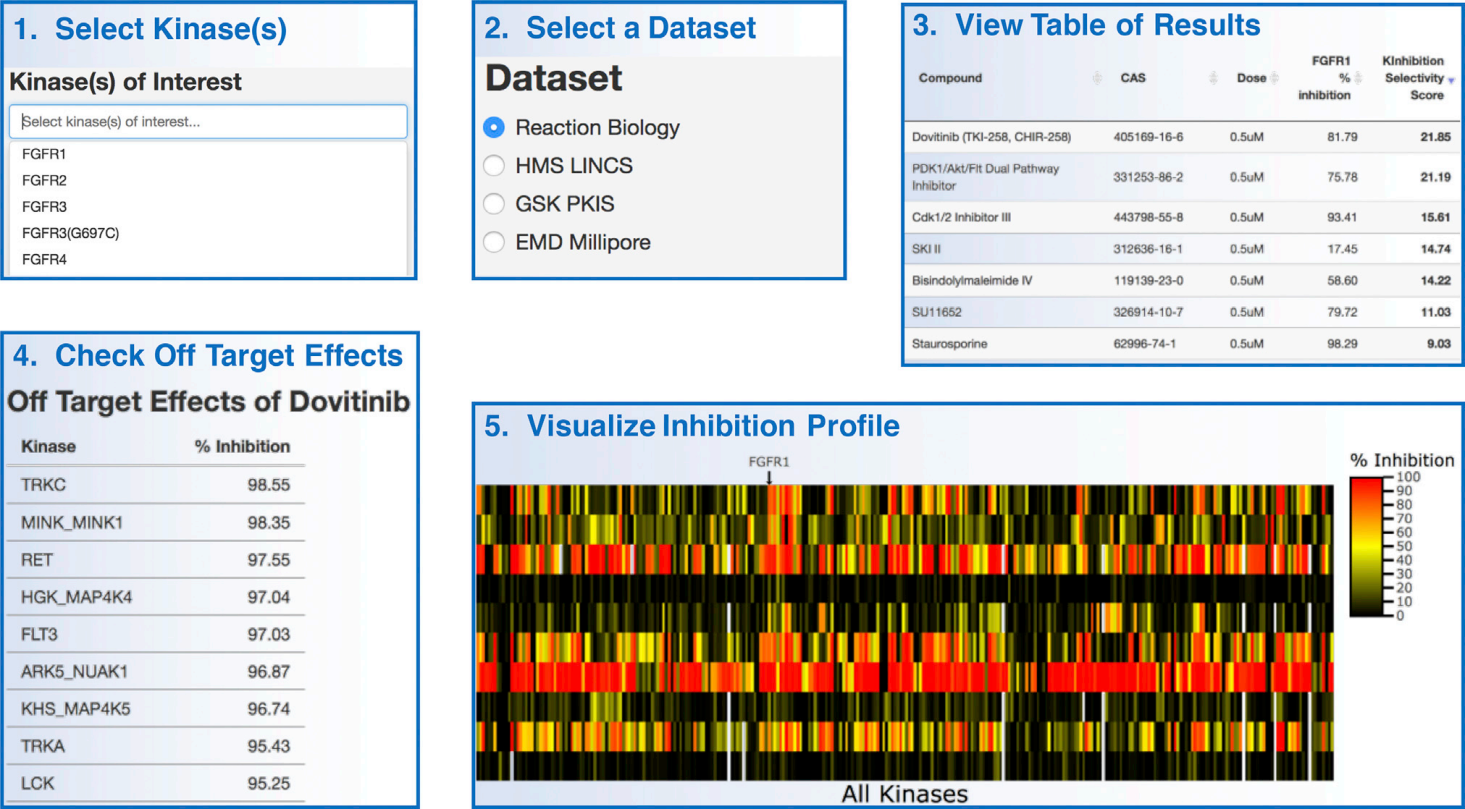
Using KInhibition Users first select a kinase or group of kinases that they wish to inhibit, and then select a dataset in which to search for compounds. The table of compounds updates and sorts compounds based on the calculated KInhibition Selectivity Score. Clicking on a row opens a second table below, which displays off-target effects of that compound. The Heatmap tab displays the full inhibition profiles across all kinases for the compounds displayed in the first page of the Table of Results. The table, and consequently the heatmap, can be reordered by clicking on any of the column headers.

The KInhibition app is run entirely in-browser and does not require the user to upload or download any data or files. It can be found at https://kinhibition.fredhutch.org/. Upon loading the app webpage, users select a kinase or a group of kinases they wish to inhibit (Figure 1). Kinase names are standardized across all datasets, as described toward the bottom of the “Datasets” tab. After selecting the kinase(s), the “Table of Results” tab will automatically update to list the inhibitors in the first available dataset, sorted based on their KInhibition Selectivity Score for the chosen kinase(s). A set of radio buttons will also appear to allow the user to choose between all the datasets that include their chosen kinase(s). The data from each dataset are kept separate and must be searched one at a time, as each screen is done using different experimental conditions, kinase panels, and compound doses, and thus results may not be comparable enough to simply merge the datasets. Details about each dataset can be found in the Datasets tab (Table 1). The Table of Results can be searched using the search box in the top right, can display more or fewer compounds per page using the drop-down menu in the top left, and can be sorted based on the values in any of the columns by clicking on the column header. Furthermore, clicking on any row generates a new table below this one, which lists the significant off-targets of that compound. “Significant off-targets” are defined as kinases inhibited at least half the amount of the chosen kinase (or half of the geometric mean for multiple chosen kinases).

**Table 1:** Informational Summary of Datasets Currently Included in KInhibition

Dataset	Compounds Tested	Compound + Dose Combinations	Kinases Screened	Pairwise Coverage	Reference
Reaction Biology^a^	178	178	300	98.9%	Anastassiadis et al., 2011
HMS LINCS	121	134	471	88.5%	Koleti et al., 2017
GSK PKIS	367	734	224	99.9%	Dranchak et al., 2013
EMD Millipore	128	255	234	100%	Gao et al., 2013

a An updated version of the Reaction Biology dataset will be included in this portal as soon as it is publicly available. Currently, the updated version contains 385 compounds, 427 compound + dose combinations, 298 kinases screened, and 99.5% pairwise coverage.

Finally, after exploring the Table of Results, users can click on the “Heatmap” tab to load a heatmap of the inhibition profiles for the compounds displayed in the Table of Results. The compounds included in the heatmap will mirror those in the first page of the Table of Results, including any changes to the number or order of compounds in this table. Inhibition is represented as a color spectrum from black (no inhibition) to yellow (moderate inhibition) to red (maximal inhibition) (Figure S1). Users can mouse over the heatmap to view the details of any individual point, or click and drag to selectively zoom in on a portion (double-clicking zooms out to the full heatmap). This heatmap, along with all the previously mentioned tables, can be downloaded using buttons below each element.

## Discussion

Kinases remain one of the few classes of biomolecules whose function (as opposed to simply concentration or abundance) can be easily detected, quantified, and perturbed. Kinases therefore sit at a critical intersection between basic research and clinical applications, making data on kinase inhibitors a lucrative asset in both academia and life science industries. With the above functionality, KInhibition fills a much needed role in modern cell biology by allowing researchers to make data-driven decisions regarding kinase inhibitors. By following the aforementioned steps, researchers can easily find and choose the most selective and appropriate compound for their particular target or pathway. Due to the robust and careful design of the app, this platform can be easily updated and expanded to include additional datasets and information as they become available. We therefore expect this portal to see broad use and adoption akin to other selection tools.

### Limitations of This Study

The KInhibition platform, and the associated KInhibition Selectivity Score were designed to best leverage the currently available data. However, it should be noted that the KInhibition Selectivity Score and all other metrics listed in this tool are based only on a single dose of the compound used. The efficacy, selectivity, and off-target effects of a given compound depend heavily on the concentration used in the actual experiment, as well as on the biological context in which the compound is applied. Thus, the information presented in the Table of Results (i.e., Percent Inhibition) may not directly translate to a cellular or *in vivo* context. Therefore, the goal of this portal, and the datasets included in it, is to obtain a qualitative assessment of selectivity, using the quantitative metrics presented as data-driven guidelines for decision making in the context of past experience and other pharmacologic properties of the compounds in question (i.e., solubility, bioavailability, and metabolism).

## Methods

All methods can be found in the accompanying Transparent Methods supplemental file.

## Data and Software Availability

The app portal can be accessed at https://kinhibition.fredhutch.org. The source code and all other files can be found at the Github repository listed in the Key Resources Table.

## Supplemental Information

Document S1. Transparent Methods and Figure S1
