# Supplementary material for: KInhibition: A Kinase Inhibitor Selection Portal
Source: iScience. 2018 Sep 18;8:49–53. doi: 10.1016/j.isci.2018.09.009 (PMC6170255; doi:10.1016/j.isci.2018.09.009)
Supplement: Document S1. Transparent Methods and Figure S1 [file mmc1.pdf]

ISCI, Volume *8*

Supplemental Information

KInhibition: A Kinase Inhibitor Selection Portal

Thomas Bello and Taranjit S. Gujral

SUPPLEMENTAL FIGURES:

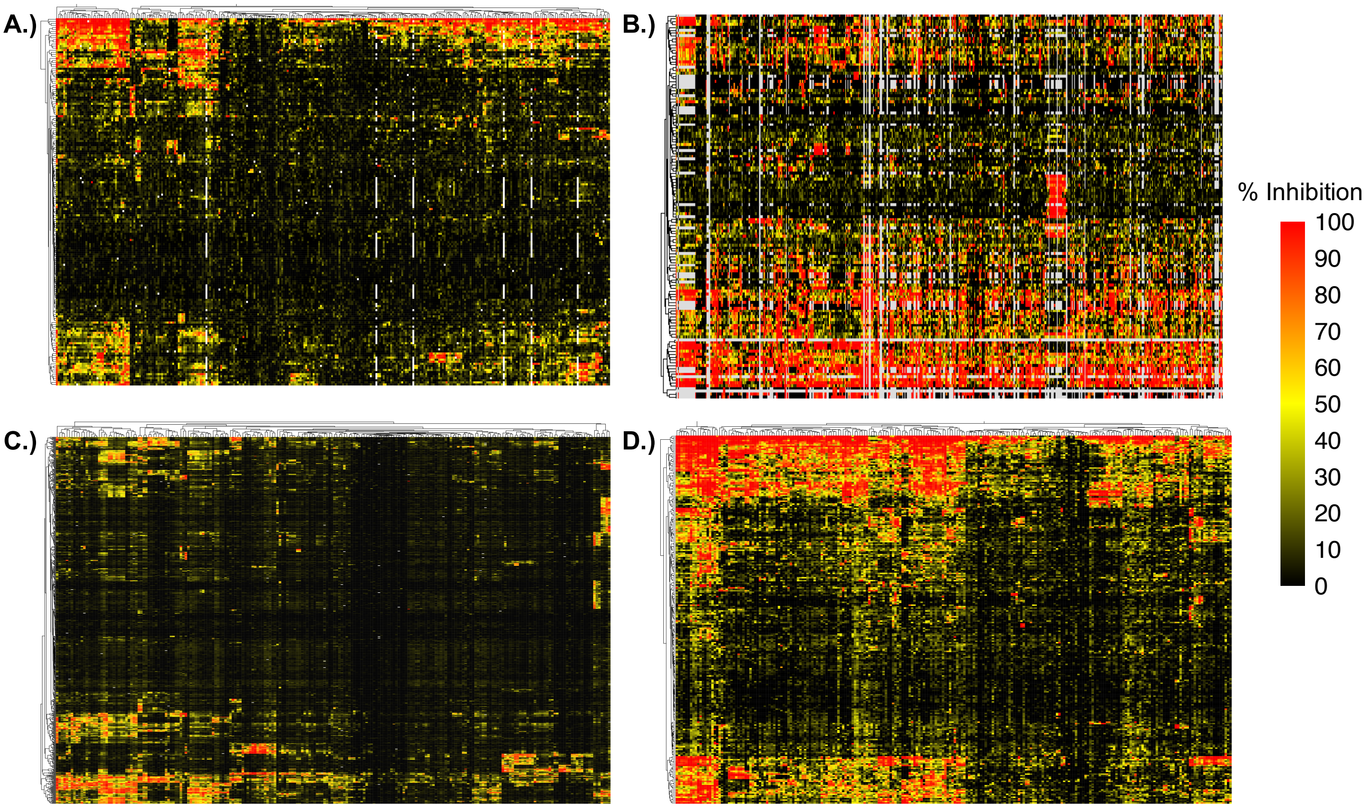

Figure S1. Heatmaps showing the inhibition profiles of all the compounds in each dataset, related to Table 1. Rows represent the compounds tested, columns represent the kinases screened. White boxes denote missing data. A, Reaction Biology dataset; B, HMS LINCS dataset; C, GSK PKIS dataset; D, EMD Millipore dataset.

TEST PROCEDURES AND VIGNETTE:

Finding a selective inhibitor of EGFR and ErbB family kinases, related to Figure 1.

To begin, navigate to https://kinhibition.fredhutch.org in a modern desktop browser (while mobile browsers may load the page, some functionality may be lost). Chrome, Firefox, and Safari have all been tested for compatibility. On loading, the sidebar on the left will have two entry fields, "Kinase(s) of Interest" and "Dataset", as well as brief instructions for use. The first step is to select a kinase or group of kinases to inhibit. In this vignette, we will search for inhibitors of EGFR and other ErbB family members. Click on the kinase selection field (labeled with the placeholder text "Select Kinase(s) of interest…"). This will open another drop-down menu with all of the kinases profiled in the chosen dataset. Select "EGFR" by either scrolling down to find it, or by typing the first few letters into the field to filter the results, then clicking on "EGFR" in the drop-down menu. Note that you must click the option even if you have typed the entire name into the field in order for the tool to register your selection. Once EGFR is selected, the Table of Results to the right will automatically populate with the top ten selective inhibitors from the first available dataset (in this case, "Reaction Biology"), initially sorted by KInhibition Selectivity Score (KISS). Additionally, a set of radio buttons will appear to allow you to select any dataset that includes the selected kinase(s) (descriptions of each dataset can be found by navigating to the "Datasets" tab). For now, let us keep "Reaction Biology" selected. If it has changed, click on the "Reaction Biology" button.

In the Table of Results, we see that the top result is "EGFR Inhibitor", with a KISS of 98.72, followed by PD 174265 with a KISS of 88.37. To the right side of the table, we notice that PD 174265 has one off-target effect that is greater than half of its inhibition of EGFR, while EGFR inhibitor has none. Note that you may need to scroll to the right to see the whole table, depending on the size of your browser window. To check this off target effect, click on the second row of the Table of Results. This will open a second table below the "Table of Results" that displays the off-target results of the selected compound (you may need to scroll down to see this table). Here, we see that PD 174265 also has significant inhibition on ErbB2 that is considered "off-target" and is lowering its KISS. To get a better idea of the full inhibition profiles of these compounds, click on the "Heatmap" tab towards the top of the screen. Note that the heatmap may take a few seconds to load, and may require scrolling to the right to see the entire image. From this heatmap, we can see that EGFR Inhibitor is, indeed, highly selective for EGFR at 0.5 μM. The white tiles represent areas of missing data, so we can see that EGFR Inhibitor was not profiled for 6 kinases in this dataset. If we hover over these tiles, a pop-up window reveals that these kinases are (from left to right) EPHA6, MYO3B, PRKACG, STK38, TLK2, and ULK3.

From the heatmap, we can see that Lapatinib (row 3) also has high inhibition of EGFR, but also inhibits two other kinases. Hovering the mouse over these bright red tiles reveals that these are ErbB2 and ErbB4. To modify our search to

include these as on-target inhibitions, first navigate back to the “Table of Results” tab. Now, click on the “Kinase(s) of Interest” field on the left again and, without removing EGFR, add “ERBB2” and “ERBB4” the same way as before (either by scrolling to find them, or typing the first few characters, then clicking on the desired kinase). With these selected, we see that Lapatinib is now the top result with a KISS of 96.31. Use the drop-down menu to the top-left of the table to change “Show 10 entries” to “Show 20 entries”. Towards the bottom of this table, we see that “EGFR Inhibitor” now has a KISS of 10.58 due to its low inhibition of ErbB2 and ErbB4. If we desire an inhibitor that has higher inhibition of ErbB4 specifically, we can click on the column name “ERBB4 % Inhibition” twice to reorder the compounds based on their inhibition of ErbB4. The top result, Bosutinib, does not appear very selective (KISS = 27.42). However, the second result is a compound named “EGFR/ErbB2/ErbB4 Inhibitor” (KISS = 87.50). Once again, we can click on this row to generate a table of the 7 notable off-target effects.

To get a well-rounded view of which inhibitor to use, it is a good idea to search multiple datasets. With the same group of kinases selected (EGFR, ERBB2, ERBB4), click on the “HMS LINCS” button under the “Datasets” header on the left. When the table loads, we see that, in this dataset, Lapatinib (10μM) has a KISS of 88.37. Clicking on the second row to display the off-target effects of Lapatinib (10μM) shows that this dataset also includes many mutant forms of EGFR that are significantly inhibited by Lapatinib. Since these are counted as off-targets, they will lower the KISS. If we wish to get a better idea of Lapatinib’s score, we can add each of these mutant forms to our “Kinase(s) of Interest). If we wish to search for a particular subset of compounds, we can type “AZ” in the search field to the top right of the table to restrict the compounds displayed to only those with “AZ” in their names. After doing this, clicking on the “Heatmap” tab will display a heatmap of only these compounds. Finally, all of these outputs (Table of Results, off-target effect table, and heatmap) can be downloaded by clicking on the buttons below each object.

TRANSPARENT METHODS:

KEY RESOURCES TABLE

REAGENT or RESOURCE	SOURCE	IDENTIFIER
**Deposited Data**		
App script	This paper	https://github.com/FredHutch/KInhibition-public (app.R)
Reaction Biology Formatted Dataset	(Anastassiadis et al., 2011)	https://github.com/FredHutch/KInhibition-public (rbio_old_dataset.csv)
HMS LINCS Formatted Dataset	(Koleti et al., 2017)	https://github.com/FredHutch/KInhibition-public (LINCS_dataset.csv)
GSK PKIS Formatted Dataset	(Dranchak et al., 2013)	https://github.com/FredHutch/KInhibition-public (PKIS_dataset.csv)
EMD Millipore Formatted Dataset	(Gao et al., 2013)	https://github.com/FredHutch/KInhibition-public (EMD_dataset.csv)
**Software and Algorithms**		
R 3.3.0	(R Core Team, 2016)	https://www.r-project.org/
Shiny	(Chang et al., 2017)	https://cran.r-project.org/package=shiny
Shiny Semantic	(Stachura, 2018)	https://cran.r-project.org/package=shiny.semantic
DT: ‘DataTables’ Wrapper	(Xie, 2018)	https://cran.r-project.org/package=DT
dplyr	(Wickham et al., 2017)	https://cran.r-project.org/package=dplyr
reshape2	(Wickham, 2007)	http://www.jstatsoft.org/v21/i12/
ggplot2	(Wickham, 2009)	http://ggplot2.org
webshot	(Chang, 2017)	https://cran.r-project.org/package=webshot
HTMLwidgets	(Vaidyanathan et al., 2018)	https://cran.r-project.org/package=htmlwidgets
plotly	(Sievert et al., 2017)	https://cran.r-project.org/package=plotly

CONTACT FOR REAGENT AND RESOURCE SHARING

Further information and requests for resources should be directed to and will be fulfilled by the Lead Contact, Taran Gujral (tgujral@fredhutch.org).

METHOD DETAILS

Calculating the KInhibition Selectivity Score

The KInhibition Selectivity Score, $KISS$, quantifies the selectivity of a given compound screened at a specific dose against a large panel of kinases. The data from such screens is treated as an $m \times n$ drug-target interaction matrix, where m is the number of compounds (with different doses of the same compound being treated as separate compounds for this purpose) and n is the number of kinases screened. All entries in this matrix fall in the range $[0, 100]$ and represent the inhibition of the kinase by that compound. This is "percent of control" data, with 0 being no inhibition (activity of the kinase equal to or greater than the control), and 100 being complete inhibition (no detected kinase activity in the presence of that compound).

Before computing the score, each row in the drug-target interaction matrix is scaled so that the maximum of every row is exactly 100. This decouples compounds' selectivity from their efficacy to avoid unnecessarily penalizing compounds that are highly selective, but display low absolute inhibition at the dose tested. Efficacy at the tested dose can still be determined in the final results table, which displays the unscaled inhibition values.

The following calculations are computed row-wise on the scaled drug-target matrix, so that each compound has an assigned selectivity score based solely on the properties of that compound, independent of the other compounds (rows) in the matrix. The non-missing elements of each row, $z_1 \dots z_n$, can be partitioned into on-target effects, $x_1 \dots x_k$, and off-target effects, $y_1 \dots y_{n-k}$. First, the Inhibition Score, IS, is computed from the k chosen on-target inhibitions $x_1 \dots x_k$ as a geometric mean:

$$IS = \sqrt[k]{\prod_{i=1}^{k} x_i}$$

We used a geometric mean to best represent compounds that inhibit only some of the chosen on-target kinases, as a geometric mean is always less than or equal to an arithmetic mean. This value thus lies in the interval of the scaled data:

$$0 \le IS \le 100$$

The Inhibition Penalty, IP, is then computed in two parts. The first part, IP_1, is computed as an arithmetic mean of the off-target effects $y_1 \dots y_{n-k}$:

$$IP_1 = \left(\frac{1}{n-k}\right) \sum_{i=1}^{n-k} y_i$$

This portion of the penalty represents the baseline inhibition across all of the off-target kinases, which is useful for penalizing compounds with extremely broad activity. However, it poorly accounts for compounds that have a small number (i.e. <10) of very large (near 100% inhibition) off-target effects compared to the on-target effects. To better capture this aspect, IP_2 is computed as a ratio of normalized variances between the off-target effects $y_1 \dots y_{n-k}$ and the total effects $z_1 \dots z_n$ as follows. First, the variances are computed as

$$s_y^2 = \left(\frac{1}{n-k-1}\right) \sum_{i=1}^{n-k} (y_i - \bar{y})$$

$$s_z^2 = \left(\frac{1}{n-1}\right) \sum_{i=1}^{n} (z_i - \bar{z})$$

where $\bar{y} = \left(\frac{1}{n-k}\right) \sum_{i=1}^{n-k} y_i$ and $\bar{z} = \left(\frac{1}{n}\right) \sum_{i=1}^{n} z_i$ are the arithmetic means of the off-target and total inhibitions, respectively. When $k \ll n$, we expect these variances to be approximately equal for all but the most selective compounds, whose total variances will instead be explained largely by the difference between the on- and off-target effects. An approximate lower bound for the off-target variance can be computed using a modified version of the von Szokefalvi Nagy Inequality:

$$s_{lo}^2 = \frac{\max(y_i)^2}{2n}$$

The variances can then be centered using this lower bound in order to shift IP_2 to have a minimum closer to 0, and IP_2 is calculated as the ratio between the two shifted variances:

$$IP_2 = \frac{s_y^2 - s_{lo}^2}{s_z^2 - s_{lo}^2}$$

While the modifications to the lower bound (namely, using n rather than $n-k$ in the denominator) mean that IP_2 can fall outside the interval of $[0, 1]$, in practice this only occurs for the most selective compounds, and then only by a very small margin. We then combine the two penalties in an empirically determined manner:

$$IP = \frac{IP_1 + 100(IP_2)^5}{2}$$

With the rare exception mentioned above, both terms in the numerator of this equation lie in the interval $[0, 100]$, and therefore their arithmetic mean IP does as well. Finally, $KISS$ is calculated as a different between IS and IP

$$KISS = IS - IP$$

Based on the bounds mentioned above, $KISS$ generally lies in the interval $[-100, 100]$, with 100 representing near perfect selectivity (i.e. only on-target inhibition), and -100 representing the opposite (i.e. only off-target inhibition).

QUANTIFICATION AND STATISTICAL ANALYSIS

All software development, calculations, and analyses were carried out using R 3.3.0 (https://www.r-project.org/). All packages used can be found in the Key Resources Table.

DATA SOFTWARE AND AVAILABILITY

The app portal can be accessed at https://kinhibition.fredhutch.org. The source code and all other files can be found at the Github repository listed in the Key Resources Table.

SUPPLEMENTAL REFERENCES:

Anastassiadis, T., Deacon, S.W., Devarajan, K., Ma, H., and Peterson, J.R. (2011). Comprehensive assay of kinase catalytic activity reveals features of kinase inhibitor selectivity. Nat. Biotechnol. *29*, 1039–1045.

Chang, W. (2017). webshot: Take Screenshots of Web Pages.

Chang, W., Cheng, J., Allaire, J.J., Xie, Y., and McPherson, J. (2017). shiny: Web Application Framework for R.

Dranchak, P., MacArthur, R., Guha, R., Zuercher, W.J., Drewry, D.H., Auld, D.S., and Inglese, J. (2013). Profile of the GSK Published Protein Kinase Inhibitor Set Across ATP-Dependent and-Independent Luciferases: Implications for Reporter-Gene Assays. PLoS One *8*, e57888.

Gao, Y., Davies, S.P., Augustin, M., Woodward, A., Patel, U.A., Kovelman, R., and Harvey, K.J. (2013). A broad activity screen in support of a chemogenomic map for kinase signalling research and drug discovery. Biochem. J. *451*, 313–328.

Koleti, A., Terryn, R., Stathias, V., Chung, C., Cooper, D.J., Turner, J.P., Vidović, D., Forlin, M., Kelley, T.T., D'Urso, A., et al. (2017). Data Portal for the Library of Integrated Network-based Cellular Signatures (LINCS) program: integrated access to diverse large-scale cellular perturbation response data. Nucleic Acids Res.

R Core Team (2016). R: A Language and Environment for Statistical Computing.

Sievert, C., Parmer, C., Hocking, T., Chamberlain, S., Ram, K., Corvellec, M., and Despouy, P. (2017). plotly: Create Interactive Web Graphics via "plotly.js."

Stachura, F. (2018). shiny.semantic: Semantic UI Support for Shiny.

Vaidyanathan, R., Xie, Y., Allaire, J.J., Cheng, J., and Russell, K. (2018). htmlwidgets: HTML Widgets for R.

Wickham, H. (2007). Reshaping Data with the {reshape} Package. J. Stat. Softw. *21*, 1–20.

Wickham, H. (2009). ggplot2: Elegant Graphics for Data Analysis (Springer-Verlag New York).

Wickham, H., Francois, R., Henry, L., and Muller, K. (2017). dplyr: A Grammar of Data Manipulation.

Xie, Y. (2018). DT: A Wrapper of the JavaScript Library "DataTables."
